# User engagement in clinical trials of digital mental health interventions: a systematic review

**DOI:** 10.1186/s12874-024-02308-0

**Published:** 2024-08-24

**Authors:** Jack Elkes, Suzie Cro, Rachel Batchelor, Siobhan O’Connor, Ly-Mee Yu, Lauren Bell, Victoria Harris, Jacqueline Sin, Victoria Cornelius

**Affiliations:** 1 Imperial Clinical Trials Unit, Imperial College London, White City Campus, Stadium House, 68 Wood Lane, London, W12 7RH UK; 2 University of Oxford, Oxford, UK; 3 Florence Nightingale Faculty of Nursing, Midwifery and Palliative Care, King’s College London, London, UK; 4 Leeds Institute of Clinical Trials Research, University of Leeds, Leeds, LS2 9JT UK; 5 City St Geroge’s, University of London, London, UK

**Keywords:** Randomised controlled trials, Digital mental health interventions, Mental health, User engagement, Digital health, Systematic review, Meta-analysis

## Abstract

**Introduction:**

Digital mental health interventions (DMHIs) overcome traditional barriers enabling wider access to mental health support and allowing individuals to manage their treatment. How individuals engage with DMHIs impacts the intervention effect. This review determined whether the impact of user engagement was assessed in the intervention effect in Randomised Controlled Trials (RCTs) evaluating DMHIs targeting common mental disorders (CMDs).

**Methods:**

This systematic review was registered on Prospero (CRD42021249503). RCTs published between 01/01/2016 and 17/09/2021 were included if evaluated DMHIs were delivered by app or website; targeted patients with a CMD without non-CMD comorbidities (e.g., diabetes); and were self-guided. Databases searched: Medline; PsycInfo; Embase; and CENTRAL. All data was double extracted. A meta-analysis compared intervention effect estimates when accounting for engagement and when engagement was ignored.

**Results:**

We identified 184 articles randomising 43,529 participants. Interventions were delivered predominantly via websites (145, 78.8%) and 140 (76.1%) articles reported engagement data. All primary analyses adopted treatment policy strategies, ignoring engagement levels. Only 19 (10.3%) articles provided additional intervention effect estimates accounting for user engagement: 2 (10.5%) conducted a complier-average-causal effect (CACE) analysis (principal stratum strategy) and 17 (89.5%) used a less-preferred per-protocol (PP) population excluding individuals failing to meet engagement criteria (estimand strategies unclear). Meta-analysis for PP estimates, when accounting for user engagement, changed the standardised effect to -0.18 95% CI (-0.32, -0.04) from − 0.14 95% CI (-0.24, -0.03) and sample sizes reduced by 33% decreasing precision, whereas meta-analysis for CACE estimates were − 0.19 95% CI (-0.42, 0.03) from − 0.16 95% CI (-0.38, 0.06) with no sample size decrease and less impact on precision.

**Discussio﻿n:**

Many articles report user engagement metrics but few assessed the impact on the intervention effect missing opportunities to answer important patient centred questions for how well DMHIs work for engaged users. Defining engagement in this area is complex, more research is needed to obtain ways to categorise this into groups. However, the majority that considered engagement in analysis used approaches most likely to induce bias.

**Supplementary Information:**

The online version contains supplementary material available at 10.1186/s12874-024-02308-0.

## Introduction

One in four people experience a mental health problem every year [1]. However, an estimated 70% with mental ill health are unable to access treatment [2]. App and web-based tools, collectively digital mental health interventions (DMHIs), are low cost, scalable [3], and have potential for overcoming traditional barriers to treatment access, such as physical access (flexibility in treatment location), confidentiality (providing anonymity), and stigma [4]. In recent years, the number of available DMHIs has rapidly increasd [5], the Apple App Store alone has over 10,000 behavioural apps [6]. This rapid increase combined with the complex nature of DMHIs has meant safety and effectiveness regulations have lagged behind [7]. Additionally, many DMHIs are developed for commercial purposes and marketed to the public without scientific evidence [8]. The current National Institute for Health and Care Excellence (NICE) guidelines [9] for digital health technologies, advocate for use of randomised controlled trials (RCTs) to evaluate the effectiveness of digital interventions in specific conditions such as mental health. Promisingly, the number of digital interventions evaluated in RCTs over the last decade has more than doubled [10].

Many DMHIs are developed through the digitalisations of existing services, such as online self-led formats of conventional therapist-delivered treatments. However, in contrary to conventional therapist-led treatments, DMHIs offer flexible anytime access for individuals [11]. This change in delivery means existing evidence of risk-benefit balance from structured therapist-delivered interventions is not translatable. DMHIs are potential solutions to provide more individuals with much needed treatment access, but they are not without challenges. In 2018 the James Lind Alliance (JLA) patient priority setting group for DMHIs set out the top 10 challenges to address [12]. Overcoming these challenges is essential for DMHIs to successfully improve treatment access and health outcomes in mental health [13, 14]. One theme that emerged from across the priorities was the importance of improving methods for evaluating DMHIs including the impact of user engagement.

The impact user engagement has on DMHIs efficacy is poorly understood [6, 15, 16]. Although DMHIs are widely available, user engagement with DMHIs is typically low [17]. For multi-component DMHIs (commonly including psychoeducation, cognitive exercises, self-monitoring diary), a minimally sufficient engagement in DMHIs is often crucial for establishing behavioural changes and thus improved health outcomes [18]. However, achieved sustained behavioural changes by engaging with DMHIs is a multidimensional construct that is both challenging to assess and the pathway for patients to achieve this is complex [19, 20]. Unlike other interventions, DMHIs are unique in that web-based or app-based interventions can capture interactions from individuals. User engagement can be measured and recorded using automatically captured indicators (e.g., pageviews, proportion of content/modules completed, or number of logins). However, the large variety in measurable indicators across different DMHIs [16, 21] further compounds challenges to understanding pathways to sustained behaviour changes.

For RCTS, the latest estimand framework in the ICH E9 R1 addendum [22] provides guidance on defining different estimands, which enables trialists to ensure the most important research questions of interest are evaluated. This includes guidance on handling post-randomisation events, such as user engagement with the DMHI, in efficacy analysis. For example, policy makers are likely to be most interested in a treatment policy estimand which provides an assessment of the benefit received on average under the new policy of prescribing the DMHI regardless of how it’s engaged with. For DMHIs typically engagement is poor, which means treatment policy estimands may underestimate the true intervention efficacy for those who engaged [23], so alternative estimands that address this may also be of interest to target. For example, the benefit received on average for individuals who would actively engage with the DMHI (a principal stratification estimand). However, to utilise available methods post-randomisation variables need to be clearly defined, but this is difficult for engagement with DMHIs because it is multifaceted with many different engagement indicators available to use.

This systematic review aimed to assess the current landscape of how RCTs for DMHIs are reported and analysed. The review primarily assessed how user engagement is described, what engagement indicators are reported and how, if at all, researchers assessed the impact of user engagement on efficacy. As the number of DMHIs evaluated in RCTs is ever increasing, this review is essential to identify current practice in trial reporting to inform further research to improve the quality of future trials. The specific research aims of interest were to: (1) examine trial design and characteristics of DMHIs; (2) summarise how user engagement had been defined and measured in RCTs of DMHIs; and (3) assess how often intervention efficacy was adjusted for user engagement and the impact of user engagement on efficacy estimates.

## Methods

The protocol for this systematic review was prospectively published in Prospero [24], and PRISMA guidance was followed in reporting of this review.

### Study selection

We included RCTs examining the efficacy of DMHIs, excluding pilot and feasibility studies [25]. Search terms for RCT designs followed guidance from Glanville et al. [26]. We included trials of participants with common mental disorders (CMD) defined by Cochrane [27] excluding populations with non-CMD comorbidities, such as patients with depression and comorbid diabetes. Populations with multiple CMDs were not excluded as there were many transdiagnostic interventions targeting overlapping symptoms of different conditions. Both trials requiring a confirmed clinical diagnosis and trials where participants self-referred were included. For consistency in DMHIs included interventions must meet any criteria from items 1.1 (targeted communication on health information), 1.3 (client to client communication, e.g., peer forums), 1.4 (health tracking or self-monitoring) or 1.6 (access to own health information) from the WHO Classification of Digital Health Interventions [28]. DMHIs must have been delivered on a mobile app or through a web-browser and where the intervention was self-guided by participants, defined as an intervention where participants have full autonomy over how this is used. Search terms for interventions followed guidance from Ayiku et al. [29]. All publications must have been reported in English.

The search was performed on the 17th September 2021 and included trials published between 1st January 2016 to 17th September 2021. Search terms were adapted for each database: MEDLINE, Embase, PsycINFO and Cochrane CENTRAL (see supplemental table S1 for search strategy). Title and abstracts were independently screened by two reviewers (JE, RB, SO, LB, LM & VH), and again at the full text review stage. Covidence [30] was used to manage all stages, remove duplicates and resolve disagreements.

### Quality

As a methodology review to examine how user engagement was described and analysed a risk of bias tool to assess trial quality was not undertaken [31]. However, key CONSORT items [32] were extracted to determine adherence to reporting guidance, including reporting of a protocol or trial registration (item 23/24), planned sample size (item 7a) and amendments to the primary analysis (item 3b). For all items self-reported data from articles was extracted.

### Data extraction

A data extraction form was developed by the lead author (JE) and reviewed by VC, SC and JS. Summary data extracted covered: trial characteristics (e.g., design and sample size); intervention and comparator descriptions (e.g., delivery method or primary function); participant demographics (e.g., age or gender); reporting of user engagement (e.g., indicators reported); and point estimates, confidence intervals and *P-values* of analysis results unadjusted and adjusted for user engagement. In trials with multiple arms the first active arm mentioned was included. No restriction was applied to the control arm in the trial. The full extraction sheet, including CONSORT items, is in the table S2 of the supplementary material.

### Analysis

The analysis was predominantly descriptive and used mean and standard deviations, or medians and interquartile ranges (IQRs) to describe continuous variables. Frequencies and percentages summarized categorical variables. User engagement captured through engagement indicators (e.g., pageviews and total logins) and methods to encourage user engagement (e.g., automatic notifications) were summarised descriptively. Indicator data was summarised in four categories: duration of use (e.g., length of session), frequency of use (e.g., number of logins), milestone achieved (e.g., modules completed) and communication (e.g., messages to therapist). Descriptive summaries also assessed both the recommended user engagement definitions, the pre-specified minimum engagement level investigators told participants to use DMHIs, and active user definitions, the pre-specified engagement level of most interest to investigators for intervention effects accounting for user engagement. Both were summarised by indicators used in definitions.

To determine the impact of user engagement on intervention efficacy, restricted maximum likelihood random effects meta-analyses were conducted for articles that reported both intervention effect when user engagement was accounted for and when it wasn’t. Standardised effects were used due to outcomes and measures varying between articles. These were taken directly, where reported, otherwise calculated using guidance from Cochrane [33], and Cohen’s d formula for the standard deviation [34]. Articles were grouped by outcome domains (e.g., depression, anxiety or eating disorders) based on the reported primary clinical outcome used to evaluate efficacy. Analyses also group articles based on the analytical approach used for adjustment, those using statistical methods that retained all participants formed one group (recommend approaches) and those using statistical methods only retaining conventional per-protocol populations, i.e., exclude the data from those who did not comply, formed the other group (per-protocol approaches). All analysis was performed using Stata 17.

## Results

**Fig. 1:**
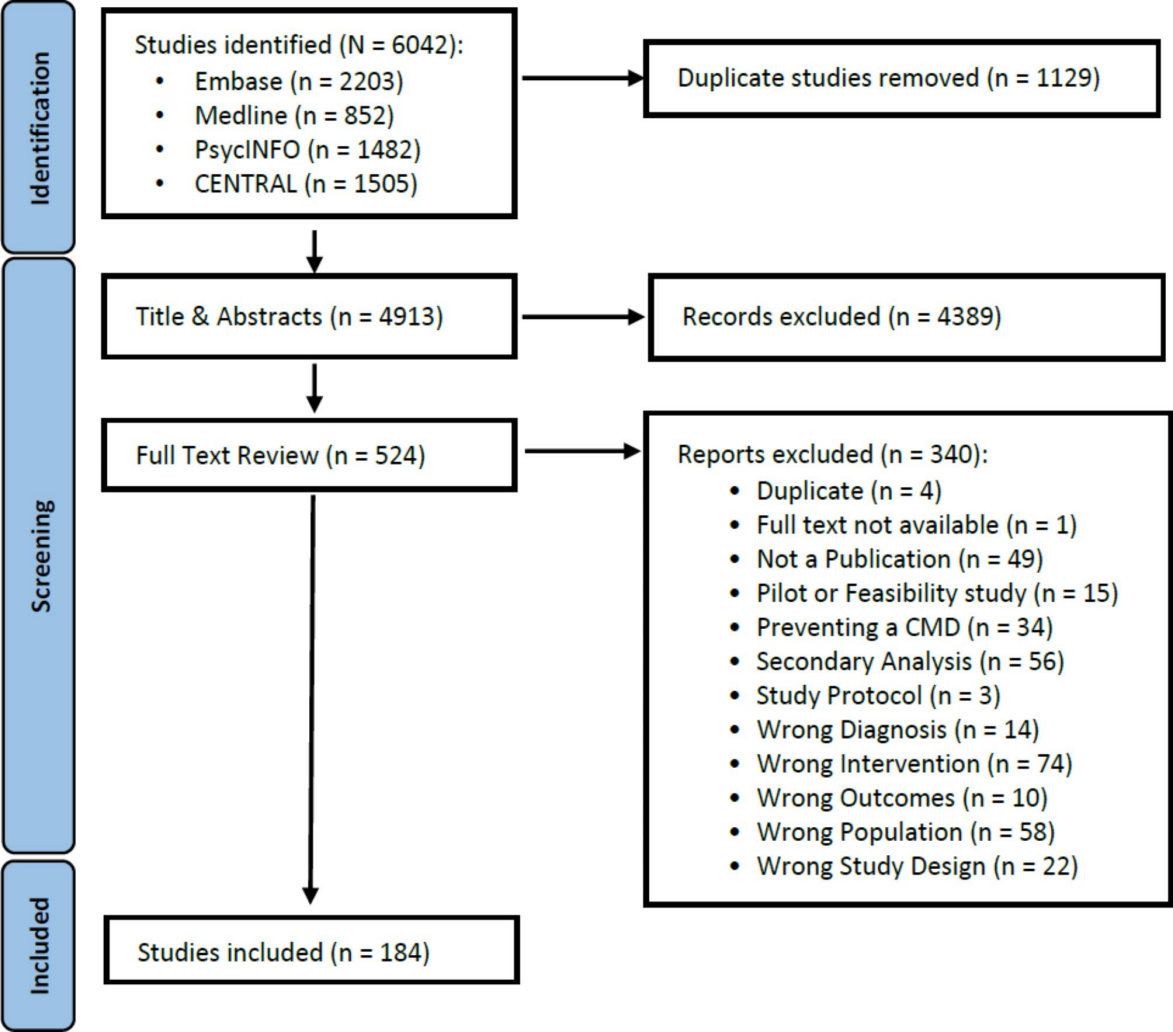
PRISMA flowchart for studies included in the systematic review

From a total of 6,042 articles identified, 184 were eligible and included in this review (see Fig. 1) randomising 43,529 participants. The most evaluated outcome domain was Depression, 74 (40.2%) articles, followed by Anxiety, 29 (15.8%) articles, and PTSD, 12 (6.5%) articles, see supplementary table S3 for full list. At least 123 unique interventions were assessed, however some interventions (*n* = 39) were only described in general terms, such as internet delivered cognitive behaviour therapy for depression, so could not be distinguished as separate interventions and are excluded from the count. On average 30.7 (SD 7.7) articles were published each year, a more detailed breakdown by outcome domain is in supplementary figures s1 and s2.

Extracted CONSORT items assessed trial reporting quality, 51 articles (27.7%) did not report their planned sample size and 36 articles (19.7%) did not clearly reference a trial protocol or trial registration number. For the 133 articles that reported both the planned and actual sample size, 43 (32.3%) failed to recruit to their target. The planned analysis approach was reportedly changed in 3 (1.6%) articles, one due to changes in the intervention [35] and the others due to high attrition [36, 37].

Most articles used “traditional” trial designs with 170 (92.4%) opting for a parallel arm design and the majority assessed only one new intervention (*n* = 134, 78.8%). Four articles (2.2%) used a factorial design allowing for the simultaneous evaluation of multiple treatments providing statistical efficiency by reducing the number of participants required in the trial. Two articles (1.1%) in the Body Dysmorphic Disorder outcome domain reported using a crossover design. However, the first had no wash-out period and instead those in the intervention arm were asked to stop engaging with the app after 16 days [38]. The second actually used a parallel arm design, where the control group received the intervention after 3 weeks [39]. Median delivery period for DMHIs was 56 days (IQR 42–84) post-randomisation and the median total follow-up time for primary outcome collection was 183 days post-randomisation (IQR 84–365).

Participants average age was 34.1 years (SD 11.1), and most participants were female (70.7%), see Table 1. Ethnicity data was not extractable in 133 (72.3%) articles. Most trials required a confirmed diagnosis of a CMD, such as through a structured interview, for inclusion (*n* = 110, 59.8%). Symptom severity could not be extracted in 97 (52.7%) trials, but where available the most common (49 trials, 56.3%) severity was a combination of both mild and moderate. Only 12 (6.5%) articles assessed participants with severe symptomatology in the depression domain (*n* = 7, 58.3%), anxiety (*n* = 1, 8.3%), psychological distress (*n* = 1, 8.3%), general fatigue (*n* = 1, 8.3%), post-traumatic stress disorder (*n* = 1, 8.3%), or psychosis (*n* = 1, 8.3%).

**Table 1:** Trial and participant characteristics for included articles

Trial Characteristics			
**Consent Process, n (%)**			
Online location (e.g., website or telephone)	119	(65.0)	
Physical location (e.g., hospital)	59	(32.2)	
Either location	5	(2.7)	
**Study Design, n (%)**			
Cluster	4	(2.2)	
Crossover	2	(1.1)	
Factorial	7	(3.8)	
Parallel	171	(92.9)	
**Trial Type, n (%)**			
Non-inferiority	5	(2.7)	
Superiority	178	(96.7)	
Unclear	1	(0.5)	
Intervention length in days, median (IQR)	56.0	(42–84)	
Primary outcome follow-up in days, median (IQR)	183	(84–365)	
Planned sample size, mean (SD)	249.8	(258.4)	
Total participants randomised, mean (SD)	236.6	(309.0)	
**Participant Characteristics**			
Age (Mean), mean (SD)	34.1	(11.1)	
Proportion of Females, mean (SD)	71.7	(19.6)	
**Ethnicity, mean proportion (SD)**			
White	70.7	(21.8)	
Black, African, Caribbean, or Black British	13.8	(15.0)	
Asian or Asian British	12.4	(19.7)	
Mixed or Multiple ethnic groups	11.3	(9.3)	
Other	9.5	(16.4)	
Not Specified	14.1	(16.0)	
Ethnicity Unclear, n studies (%)	133	(72.3)	
**Diagnosis Reporting, n studies (%)**			
Clinical Diagnosis	110	(59.8)	
Either	27	(14.7)	
Self-referred	44	(23.9)	
Unclear	3	(1.6)	
**Top 5 Primary Outcome Diagnosis**,** n studies (%)**			
Anxiety	29	(15.8)	
Depression	74	(40.2)	
Distress	11	(6.0)	
Eating Disorders	9	(4.9)	
PTSD	12	(6.5)	

Most interventions were delivered through a website, 145 (78.8%), see Table 2. There were 76 (41.3%) trials that adapted interventions from existing in-person therapist led interventions, and 84 (45.7%) interventions were newly developed. App delivered interventions were more likely to be newly developed, 23 (71.9%), compared to website interventions, 57 (39.3%). Most common choice of control arm was usual care, 126 (68.5%). For articles with usual care as control, most opted to use wait-lists, 94 (74.6%), where intervention access was provided either immediately after the intervention period, 62/94 (66.0%), or after the total follow-up period, 32/94 (34.0%).

**Table 2:** The types of DMHI and comparators included

Intervention Description		
**Delivery Method, n (%)**		
Through an App	32	(17.4)
Website/Online	146	(79.3)
Both	6	(3.3)
**Intervention Therapeutic Focus, n (%)**		
Diagnosis Only	142	(78.0)
Transdiagnostic (i.e. to treat several Diagnosis)	36	(19.8)
Wellbeing (i.e. more generalised mental health support)	4	(2.2)
**Intervention Origin, n (%)**		
Adapted from existing in-person intervention	76	(41.3)
Original (developed for DMHI)	84	(45.7)
Unclear	24	(13.0)
Intervention Features*, n studies (%)		
Provides remote education (Structured or Unstructured)	151	(82.1)
Provides online therapy (e.g. CBT)	157	(85.3)
Symptom tracking available	42	(23.0)
Guided virtual environment	15	(8.2)
Virtual access to professional help	71	(38.6)
Online forum for patients (with other patients or professional)	15	(8.2)
**Control Comparator Type, n (%)**		
Alternative DMH Intervention	11	(6.0)
Attention control (fake version of DMHI)	35	(19.0)
In-person equivalent	12	(6.5)
Standard of Care / Treatment as Usual	126	(68.5)
**Are those in Control Comparator arm ever offered the Primary Intervention?, n (%)**		
No	89	(48.6)
Yes	94	(51.4)
**If Yes above – When are they offered the Primary Intervention?, n (%)**		
After Primary Outcome Follow-up Period	32	(34.0)
After Intervention Period	62	(66.0)

* - Denotes studies can be in more than one category

Most articles, 136 (73.9%), reported using at least one approach to encourage participants to engage with the intervention. Methods of encouragement were automatic notifications, *n* = 49/136 (32.5%), contacting participants by telephone or email, *n* = 68/136 (45.0%), or automated feedback on homework exercises, *n* = 76/136 (50.3%). Most used only one method of encouragement, *n* = 85 (62.5%), with 6 (4.4%) articles using all 3 methods of encouragement. Although many articles encouraged engagement, only 23.9% (*n* = 44) provided a recommended level of engagement to participants. Recommendations varied from using a rate to progress through content (e.g., one module per week or maximum of two modules per week), a specified duration to use the intervention (e.g., 1.5 h per week or 4 to 6 h per week), or specifying milestones to complete (e.g., complete one lesson every 1–2 weeks or complete daily homework assignments), a full list is in table s5 of the supplementary material.

**Fig. 2:**
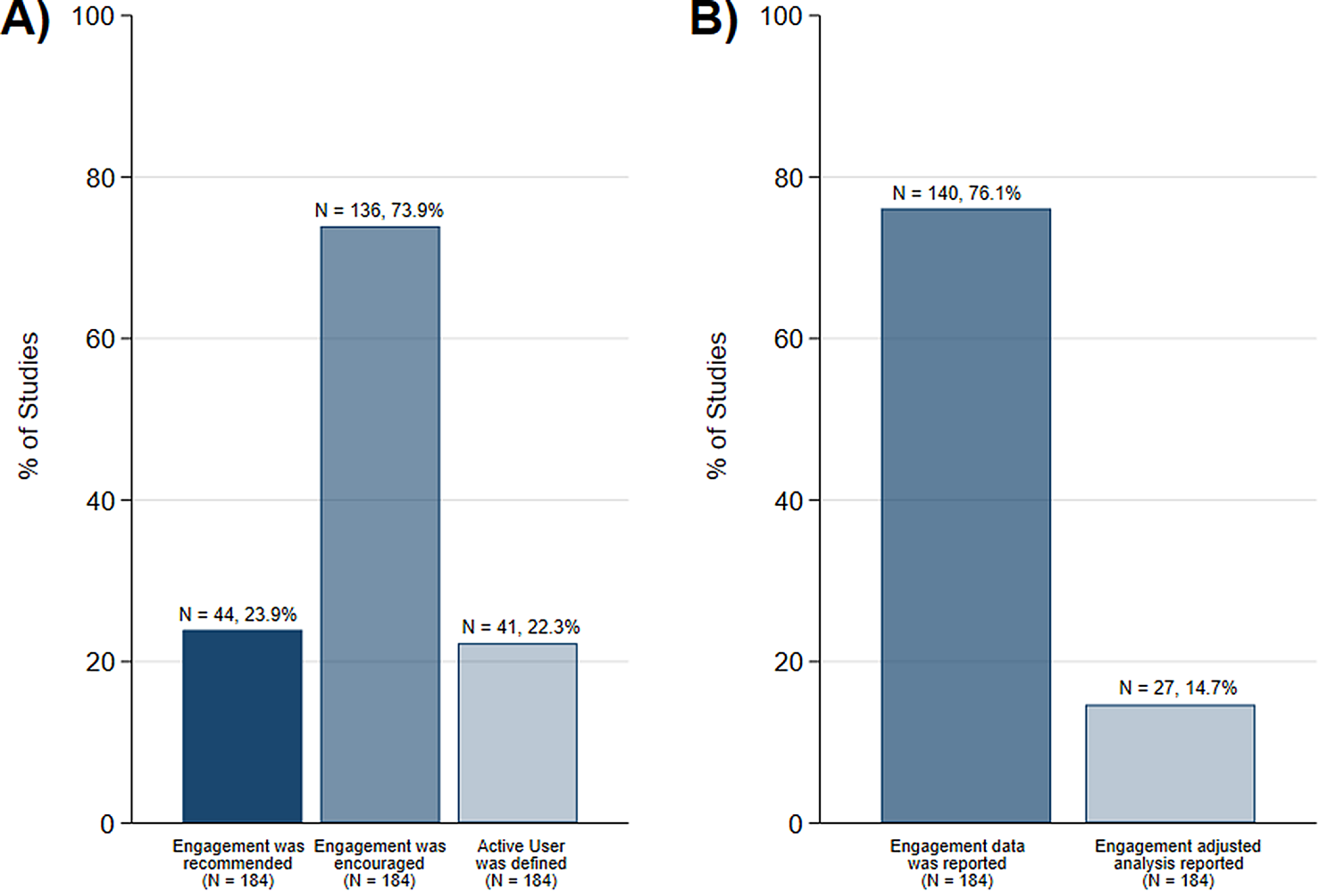
Proportion of trials describing user engagement in methods section (**A**) or in results section (**B**) A) – How user engagement was reported in the methods section **Recommended** – the participant was told how to use the intervention by the study team **Encouraged** – reminders (e.g., notifications or emails) were sent to the participant **Active User** – participants meeting a pre-specified engagement level set by the study team B) – How user engagement data was reported in the results section **Reported** – results describe activity for at least one engagement indicator **Analysis** – results report an intervention effect where user engagement has been considered

User engagement data captured through indicators was reported in many articles, 76.1% (*n* = 140), Fig. 2. Typically, this included only reporting only one indicator (*n* = 41, 29.3%) ranging up to eight indicators for one (0.7%) trial [40]. Across the 140 studies reporting user engagement data, most commonly indicators described the frequency of use, 150 (40.7%), followed by indicators to capture milestones achieved, 124 (33.6%), further detail is found in table s4 of the supplemental. A total of 150 unique indicators were reported across the 140 articles, the most popular measure used was modules completed, 51.3% (*n* = 77), followed by the number of logins, 25.3% (*n* = 38). In website only delivered interventions there were 102 unique indicators compared to 41 unique indicators reported in app-based interventions, and 7 unique indicators in interventions delivered as both an app and website.

*Active user* definitions, the engagement level of most interest to trial teams, was stated in the methods sections for 20.1% (*n* = 37) of articles. Digital components of active user definitions included setting a minimum number of modules completed (e.g., 4 out of 5 modules), a proportion of content accessed (e.g., at least 25% of pages viewed), or the total time accessed (e.g., used app for 30 min per week), a full list of active user definitions is in table s6 of the supplemental. From the 37 articles reporting active user definitions, 27 (14.7%) described statistical methods to perform an analysis accounting for user engagement but only 19 (10.3%) reported intervention effect estimates.

All articles reporting effects from the analysis accounting for user engagement also reported effects not accounting for engagement so were included a meta-analysis, Table 3. All articles used a treatment policy estimand (including all participants randomised regardless of the level of user engagement) for their primary outcome, where user engagement was not accounted for. In articles reporting an analysis accounting for user engagement, all outcome domains reported an increase in overall effect size favouring the intervention in comparison to estimates from analysis not accounting for user engagement. The largest increase in intervention efficacy was in the distress domain (*n* = 1) where the standardised mean effect size increased from − 0.61 (95% CI -0.86 to -0.36) to -0.88 (95% CI -1.17 to -0.59).

**Table 3:** Comparison of unadjusted and adjusted (for engagement) estimated intervention effects by outcome domain

Domain Analysis	No. of Studies	Unadjusted Analysis			Engagement Adjusted Analysis	
		Total Sample Size	Standardised Effect Size (95% CI)		Total Sample Size	Standardised Effect Size (95% CI)
Depression	11	3604	-0.16 (-0.23 to -0.09)		3051	-0.18 (-0.26 to -0.11)
Distress	1	262	-0.61 (-0.86 to -0.36)		207	-0.88 (-1.17 to -0.59)
OCD	1	96	-0.03 (-0.43 to 0.37)		82	-0.06 (-0.50 to 0.38)
Panic	1	142	0.03 (-0.30 to 0.36)		56	-0.14 (-0.67 to 0.39)
Relapse	3	944	0.16 (-0.14 to 0.46)		752	0.15 (-0.16 to 0.47)
Remission	2	1266	0.07 (-0.26 to 0.40)		1127	-0.00 (-0.38 to 0.38)

Note – Negative effect sizes favour the intervention, and positive effect sizes favour the control

The results comparing changes in the intervention effect by the analysis approach used (recommended versus per-protocol) is in Table 4. From the 19 articles included in the analysis, 17 (89.5%) used a conventional per-protocol (i.e., exclude the data from those who did not comply) approach for the analysis accounting for user engagement [41]. A consequence of which is that the average sample size decreased to 76.9% (IQR 67.7–87.6%) of the original size, in the active arm the average size decreased by 61.8% (IQR 38.1–75.4%). The overall standardised intervention effect increased from − 0.14 (95% CI -0.24 to -0.03, *n* = 17), *p* = .01, to -0.18 (95% CI -0.32 to -0.04, *n* = 17), *p* = .01, but was also less precise. Two trials used a Complier Average Causal Effect (CACE) analysis [42], a recommended approach where assumptions hold, with all participants randomised included in the analysis. The overall standardised intervention effect increased in the meta-analysis with an overall change from − 0.16 (95% CI -0.38 to 0.06, *n* = 2), *p* = .16, to -0.19 (95%CI -0.42 to 0.03, *n* = 2), *p* = .09, with no decrease in sample size and slightly less impact on the precision of the estimate.

**Table 4:** Comparison of unadjusted and adjusted (with engagement) estimated intervention effect between analysis approaches

Analysis Approach Publication	No. of Studies	Unadjusted Analysis			Engagement Adjusted Analysis	
		Total Sample Size	Standardised Effect Size (95% CI)		Total Sample Size	Standardised Effect Size (95% CI)
**Per-Protocol Approaches**						
McCloud 2020		168	-0.21 (-0.51 to 0.09)		116	-0.14 (-0.55 to 0.27)
Gladstone 2018*		369	-0.35 (-5.64 to 4.94)		245	-0.45 (-4.34 to 3.43)
Schroder 2020		96	-0.03 (-0.43 to 0.37)		82	-0.06 (-0.50 to 0.38)
Christoforou 2017		142	0.03 (-0.30 to 0.36)		56	-0.14 (-0.67 to 0.39)
Kenter 2016		269	0.04 (-0.20 to 0.28)		182	0.03 (-0.29 to 0.36)
Braun 2021		340	-0.12 (-0.33 to 0.10)		218	-0.21 (-0.56 to 0.14)
Heckendorf 2019		262	-0.61 (-0.86 to -0.36)		207	-0.88 (-1.17 to -0.59)
Nilsson 2019		153	0.00 (-0.41 to 0.41)		134	0.01 (-0.67 to 0.68)
Mohr 2019		270	0.01 (-0.22 to 0.25)		204	0.00 (-0.27 to 0.28)
Beevers 2017		376	-0.38 (-0.62 to -0.14)		306	-0.40 (-0.65 to -0.16)
Klein 2017*		1013	0.15 (-4.59 to 4.89)		977	0.17 (-4.74 to 5.08)
DeZwaan 2017		169	0.17 (-0.14 to 0.47)		153	0.16 (-0.16 to 0.48)
Mantani 2017		164	-0.30 (-0.61 to 0.00)		117	-0.21 (-0.58 to 0.15)
Jacobi 2017*		253	0.07 (-0.26 to 0.40)		150	-0.00 (-0.38 to 0.38)
Buntrock 2016*		406	-0.29 (-2.51 to 1.93)		354	-0.19 (-3.51 to 3.13)
Lobner 2018		542	-0.16 (-0.33 to 0.01)		488	-0.17 (-0.34 to 0.01)
Klein 2016		1013	-0.19 (-0.32 to -0.07)		977	-0.20 (-0.33 to -0.08)
**Overall**	17	6005	-0.14 (-0.24 to -0.03)		4966	-0.18 (-0.32 to -0.04)
**Recommended Approaches**						
Castro 2020		111	-0.17 (-0.54 to 0.20)		111	-0.19 (-0.56 to 0.19)
Montero-Marin 2016		198	-0.16 (-0.44 to 0.12)		198	-0.20 (-0.48 to 0.08)
**Overall**	2	309	-0.16 (-0.38 to 0.06)		309	-0.19 (-0.42 to 0.03)

* - study originally reported a binary outcome, standardised effects calculated using formula from Chinn 2000

Note – Negative effect sizes favour the intervention, and positive effect sizes favour the control

## Discussion

This systematic review found that in trials of DMHIs for CMDs, promisingly many articles reported user engagement as summaries of automatically captured indicators, but the reported intervention effect rarely accounted for this. Overall, trials were not well reported, almost 30% did not reference a trial protocol and only 27% of articles had available data on ethnicity. The JLA patient priority group set user engagement as a research priority in 2018 and this review, including publications between 2016 and 2021, supports evidence that engagement data has been poorly utilised where only 10% (*n* = 19) of articles had available estimates to evaluate the impact of user engagement on intervention efficacy. Many (> 70%) articles reported summarised engagement data highlighting plenty of opportunities to better utilise this data and understand the relationship between user engagement and efficacy, a question of particular interest to the individual using DMHIs to know the true intervention efficacy.

Many articles reported at least one method used to encourage participants to engage with the intervention, however very few articles were able to specify what the recommended level of engagement should be for individuals. Additionally, only a small proportion of trials assessed the impact of user engagement on the intervention efficacy through *active user* definitions, but these were broad ranging and used a variety of different engagement indicators. This highlights the complex and challenging task to properly assess user engagement where currently there is little guidance available. This also shows how difficult it is for researchers to identify what the minimum required engagement to the intervention, active user definitions, should be due to the heterogeneity in both the individuals being treated and how the intervention is being delivered (e.g., timeliness and access to other support).

Most articles performing an analysis that accounted for engagement used a conventional per-protocol population. Although the per-protocol population can be unbiased under the strong assumption that user engagement is independent from treatment allocation [43], typically use of this population causes bias in the estimated intervention effect [44] and the underlying estimand cannot be determined, i.e. unclear precisely what is being estimated. User engagement is a post-randomisation variable and the estimand framework [22] suggests using more appropriate strategies for handling post-randomisation events. For example, conducting a complier average causal effect analysis [42] under the principal stratification strategy estimated using instrumental variable regression [45] with randomised treatment allocation used as the instrumental variable. Alternative statistical methods can also be used to implement the estimand framework [46], but due to large variation in the reported engagement indicators and therefore difficulties in how engagement as a post-randomisation variable should be defined comparisons between trials remain challenging.

Establishing better methods in how user groups are defined, based on all available engagement measures, for example by using clustering algorithms combining all engagement measures, are needed. Secondly, once groups are defined existing statistical methods available to implement the estimand framework need to be assessed to determine the optimal approach to analyse the impact of engagement on the efficacy analysis. This is now the focus of our future work.

### Future implications

The JLA priority setting partnership occurred in 2018, meaning this review of publications between 2016 and 2021, includes very few trials recruiting after 2018. Therefore, implementation of the JLA priorities cannot be assessed. However, this review has shown user engagement data was available, showing potential for more trials to explore engagement in efficacy analysis. An update of this systematic review should be performed for the next 5 years (2021–2026) to assess whether issues identified in this review around user engagement have been improved. More trials exploring engagement in efficacy analysis will mean the pathway of sustained behaviour changes through engagement with DMHIs is better understood. Additionally, reporting of user engagement varied greatly, and although the CONSORT extension of e-health [47] outlines some detail on engagement reporting, more directed guidance is needed. Improvements should include reporting what and how many indicators were available and better guidance on how indicator data should be summarised. Additionally, trial publications varied greatly in quality of reported results and particularly for key demographic information such as ethnicity. CONSORT trial reporting guidance has been around since 1996 and more journals should enforce its implemented to ensure robust reporting of trials.

Finally, where data was available, participants were mostly female, white ethnicity and young, demographics consistent with another systematic review of DMHI trials [48] and the most recent 2014 Adult Psychiatric Morbidity Survey (APMS) for who is most likely to receive treatment [49]. However, the APMS 2014 also shows that individuals from black or mixed ethnicities are more likely to experience a CMD than those from white ethnicities. This supports other literature [50, 51] and highlights differences in those recruited into trials and those who experience a CMD and not represented in DMHI efficacy estimates.

### Strengths and limitations of the review

This systematic review assessed a wide-ranging number of outcome domains, providing an overview for all current DMHIs evaluated, including articles from CMDs with lots of active research, such as anxiety and depression, to CMDs with very few published results. Additionally, this review collected detailed information on engagement indicators, how these were reported, and how they were utilised in the analysis of the intervention effect, providing a rich database of the typical indicators available across a wide range of DMHIs.

As the focus of this review was to assess user engagement the review does not analyse the temporal differences of when primary outcome data for the intervention effect were collected. This means the review ignores that differences of the intervention effects across articles could partly be due to temporal differences in when they were collected, assuming the intervention effect changes over time. However, comparisons of adjusted and unadjusted intervention effects are measured at the same timepoints within each article. Additionally, as very few studies reported analysis adjusted for user engagement there was limited data to assess the impact of user engagement on the intervention efficacy in most outcome domains. Further, as most studies assessing engagement used a similar approach, per-protocol population, a formal comparison of methods was not possible. Finally, as this review only focused on appraising how engagement was reported and statistical methods used to analyse engagement, we don’t consider the impact of loss to follow-up has on the efficacy of interventions but must acknowledge that DMHIs typically have high drop-out rates from studies with very low proportions of individuals completing the intervention [52].

## Conclusion

This review assessed reporting of user engagement and how authors considered engagement in the efficacy analysis of digital mental health interventions. While many articles reported at least one measure of engagement, very few articles used the data to analyse how engagement affects intervention efficacy, making it difficult to draw conclusions on the impact of engagement. In the small proportion of articles that reported this analysis, nearly all used statistical methods at high risk of bias. There is a clear need to improve the methods used to define active users by using all available engagement measures. This will help ensure a more consistent approach to how user engagement as a post-randomisation variable is defined. Once these methods are established trialists can then utilise existing statistical methods to target alternative estimands, such as principal stratification, that mean the impact of user engagement with the intervention efficacy can be explored.

### Electronic supplementary material

Below is the link to the electronic supplementary material.

Supplementary Material 1

## Data Availability

The study protocol is already available on Prospero (CRD42021249503), datasets used are available from the corresponding author on reasonable request after the NIHR fellowship from which this project comes from is completed (April 2025). Any researchers interested in using the data extracted can contact the lead author using the shared correspondence information.
