# Supplementary material for: User engagement in clinical trials of digital mental health interventions: a systematic review
Source: BMC Med Res Methodol. 2024 Aug 24;24:184. doi: 10.1186/s12874-024-02308-0 (PMC11344322; doi:10.1186/s12874-024-02308-0)
Supplement: Supplementary file 1 — Supplementary Material 1 [file 12874_2024_2308_MOESM1_ESM.docx]

Supplemental

Table S1 – Example Search string from Cochrane CENTRAL

| **ID** | **Search** | **Hits** |
| --- | --- | --- |
| #1 | MeSH descriptor: [Adjustment Disorders] explode all trees | 251 |
| #2 | MeSH descriptor: [Mood Disorders] explode all trees | 13482 |
| #3 | MeSH descriptor: [Affective Symptoms] explode all trees | 461 |
| #4 | MeSH descriptor: [Anorexia Nervosa] explode all trees | 553 |
| #5 | MeSH descriptor: [Anxiety Disorders] explode all trees | 7293 |
| #6 | MeSH descriptor: [Suicide, Attempted] explode all trees | 445 |
| #7 | MeSH descriptor: [Bipolar Disorder] explode all trees | 2764 |
| #8 | MeSH descriptor: [Bulimia] explode all trees | 541 |
| #9 | MeSH descriptor: [Fatigue Syndrome, Chronic] explode all trees | 403 |
| #10 | MeSH descriptor: [Combat Disorders] explode all trees | 131 |
| #11 | MeSH descriptor: [Conversion Disorder] explode all trees | 43 |
| #12 | MeSH descriptor: [Depression] explode all trees | 13147 |
| #13 | MeSH descriptor: [Depressive Disorder] explode all trees | 12759 |
| #14 | MeSH descriptor: [Dysthymic Disorder] explode all trees | 181 |
| #15 | MeSH descriptor: [Feeding and Eating Disorders] explode all trees | 1725 |
| #16 | MeSH descriptor: [Sexual Dysfunctions, Psychological] explode all trees | 2180 |
| #17 | MeSH descriptor: [Hypochondriasis] explode all trees | 96 |
| #18 | MeSH descriptor: [Hysteria] explode all trees | 19 |
| #19 | MeSH descriptor: [Erectile Dysfunction] explode all trees | 1507 |
| #20 | MeSH descriptor: [Mental Disorders] explode all trees | 76985 |
| #21 | MeSH descriptor: [Munchausen Syndrome] explode all trees | 2 |
| #22 | MeSH descriptor: [Neurasthenia] explode all trees | 29 |
| #23 | MeSH descriptor: [Neurotic Disorders] explode all trees | 301 |
| #24 | MeSH descriptor: [Obsessive-Compulsive Disorder] explode all trees | 1090 |
| #25 | MeSH descriptor: [Panic Disorder] explode all trees | 967 |
| #26 | MeSH descriptor: [Phobic Disorders] explode all trees | 1405 |
| #27 | MeSH descriptor: [Stress Disorders, Post-Traumatic] explode all trees | 2800 |
| #28 | MeSH descriptor: [Stress, Psychological] explode all trees | 6474 |
| #29 | MeSH descriptor: [Seasonal Affective Disorder] explode all trees | 175 |
| #30 | MeSH descriptor: [Self-Injurious Behavior] explode all trees | 1562 |
| #31 | MeSH descriptor: [Self Mutilation] explode all trees | 37 |
| #32 | MeSH descriptor: [Somatoform Disorders] explode all trees | 741 |
| #33 | (1 OR 2 OR 3 OR 4 OR 5 OR 6 OR 7 OR 8 OR 9 OR 10 OR 11 OR 12 OR 13 OR 14 OR 15 OR 16 OR 17 OR 18 OR 19 OR 20 OR 21 OR 22 OR 23 OR 24 OR 25 OR 26 OR 27 OR 28 OR 29 OR 30 OR 31 OR 32) | 1819488 |
| #34 | MeSH descriptor: [Internet-Based Intervention] explode all trees | 236 |
| #35 | MeSH descriptor: [Smartphone] explode all trees | 460 |
| #36 | MeSH descriptor: [Mobile Applications] explode all trees | 888 |
| #37 | MeSH descriptor: [Mobile Applications] explode all trees | 888 |
| #38 | smartphone OR App OR Website | 14182 |
| #39 | (34 OR 35 OR 36 OR 37 OR 38) | 466585 |
| #40 | MeSH descriptor: [Clinical Trials as Topic] explode all trees | 48465 |
| #41 | MeSH descriptor: [Adaptive Clinical Trials as Topic] explode all trees | 0 |
| #42 | MeSH descriptor: [Clinical Trial] explode all trees | 141 |
| #43 | MeSH descriptor: [Randomized Controlled Trials as Topic] explode all trees | 15003 |
| #44 | MeSH descriptor: [Randomized Controlled Trial] explode all trees | 119 |
| #45 | MeSH descriptor: [Controlled Clinical Trials as Topic] explode all trees | 15155 |
| #46 | MeSH descriptor: [Pragmatic Clinical Trials as Topic] explode all trees | 29 |
| #47 | (Clinical Trial or Randomised Clinical Trial OR RCT):ti,ab,kw | 644638 |
| #48 | (40 OR 41 OR 42 OR 43 OR 44 OR 45 OR 46 OR 47) | 588930 |
| #49 | 33 AND 39 AND 48 | 8598 |
| **Date Run: 17/09/2021 11:10:57** | | |

Table S2 – Data Extraction Template

| **Component** | **Variables** | **Values** |
| --- | --- | --- |
| Trial Characteristics | **ID** |  |
|  | **Year of Publication** |  |
|  | **First Author** |  |
|  | **Advertisement Strategy** | Categorical: Physical Sites, Digital Site, Both |
|  | **Recruitment Strategy** | Categorical: Physical Sites, Digital Site, Both |
|  | **No. of Arms** |  |
|  | **Study Design** | Categorical: Pilot, Feasibility, Parallel, Crossover, Cluster, Factorial, etc. |
|  | **Trial Type** | Categorical: Superiority, Non-inferiority, Other. |
|  | **Study Duration - Intervention Follow-up** |  |
|  | **No. Participants Randomised** |  |
| CONSORT Items | **Is a published protocol/trial number provided?** | Categorical: Yes or No |
|  | **No. Planned Participants (Sample Size)** |  |
|  | **Was the primary analysis changed?** | Categorical: Yes or No |
| Intervention and Comparator Descriptions | **Intervention Name** |  |
|  | **More Detail in the Protocol?** | Categorical: Yes or No |
|  | **Intervention Delivery Method** | Categorical: Website, App, Other |
|  | **Intervention Type** | Categorical: Virtual World, Structured Education, Online Therapy (e.g. Behavioural), Virtual Supprot Agent, Automated Chat Bot, Decision Aid |
|  | **Intervention Components** | Categorical: Digital Only, Blended Approach |
|  | **Intervention Support** | HEADING ONLY |
|  | **F2F Session** | Categorical: Yes or No |
|  | **Telephone Communication** | Categorical: Yes or No |
|  | **Group Delivery** | Categorical: Yes or No |
|  | **Intervention Purporse** | HEADING ONLY |
|  | **For Prevention of Symptoms?** | Categorical: Yes or No |
|  | **For Treatment?** | Categorical: Yes or No |
|  | **For Self-management?** | Categorical: Yes or No |
|  | **Intervention Focus** | Categorical: Disease Specific or Transdiagnostic |
|  | **Intervention Primary Function** | HEADING ONLY |
|  | **Promote Behavioural Change** | Categorical: Yes or No |
|  | **Encourage Social Connection** | Categorical: Yes or No |
|  | **Improve disease knowledge** | Categorical: Yes or No |
|  | **Intervention Features** | HEADING ONLY |
|  | **Remote Education** | Categorical: Yes or No |
|  | **Symptom Tracking** | Categorical: Yes or No |
|  | **Access to Professionals** | Categorical: Yes or No |
|  | **Forum for Patients** | Categorical: Yes or No |
|  | **Intervention Origin** | Categorical: Adapted, New or Unclear |
|  | **Does the intervention allow collection of trial outcomes?** | Categorical: Yes or No |
|  | **Did the intervention provide therapy for the patients carer or family?** | Categorical: Yes or No |
|  | **Was it reported that the intervention changed during the trial?** | Categorical: Yes or No |
|  | **Comparator Type** | Categorical: Placebo, Standard Care, Wait-List or Alternative DHMI |
|  | **Are those in Comparator Arm ever offered intervention?** | Categorical: Yes or No |
|  | **If Yes - When are they offered the intervention?** | Categorical: After Intervention Period, After Follow-up, Unclear. |
| Demographics | **Diagnosis Studied** | Categorical: Adjustment Disorder, Affective Disorders, Anorexia Nervosa, Anxiety, Attempted Suicide, Bipolar Disorder…. |
|  | **Diagnosis Severity** | Categorical: Mild, Moderate, Severe, Multiple, Self-Referred, Unclear |
|  | **Diagnosis Reporting** | Categorical: Self-referred, Clinically Diagnosed, Both, Unclear |
|  | **Population Setting** | Categorical: Hospital, GP, Community, School or University, Unrestricted, Other |
|  | **Age Category** | Categorical: Child, Adolescent, Young Adult, Adult, Elderly |
|  | **Age Mean** |  |
|  | **Age SD** |  |
|  | **Proportion Female** |  |
|  | **Ethnicity Included** |  |
| Engagement Reporting | **Intervention access** | Categorical: Restriced, unrestricted, unclear, Not Applicable |
|  | **Intervention Delivery Pace** | Categorical: Self-guided, Only when needed, Clinically Managed, Externally Managed, Automated, Unclear |
|  | **What methods were used to encourage participants engagement?** | HEADING ONLY |
|  | **Automatic notifications** | Categorical: Yes or No |
|  | **Reminders by clinical team** | Categorical: Yes or No |
|  | **Blended intervention (F2F component)** | Categorical: Yes or No |
|  | **Inclusion of Homework Tasks** | Categorical: Yes or No |
|  | **Other** |  |
|  | **Was engagement recommended?** | Categorical: Yes or No |
|  | **If yes - what was the definition used?** | |
|  | **If yes - No. that achieved recommendation** | |
|  | **Was engagement described?** | Categorical: Yes or No |
|  | **How was engagement desrcibed?** | HEADING ONLY |
|  | **In text?** | Categorical: Yes or No |
|  | **Using Tables** | Categorical: Yes or No |
|  | **Using Figures** | Categorical: Yes or No |
|  | **Indicators used in description** |  |
|  | **First Engagement Summary Method** | Free Text Option |
|  | **Engagement Summary Statistic** | Categorical: Mean, Median, Other |
|  | **Location Estimate** |  |
|  | **Range Estimate** |  |
|  | **Second Engagement Summary Method** | Free Text Option |
|  | **Engagement Summary Statistic** | Categorical: Mean, Median, Other |
|  | **Location Estimate** |  |
|  | **Range Estimate** |  |
|  | **Other Engagement Information** | Free Text Option, Copy from Text |
|  | **Engagment Summary (Binary)** | HEADING ONLY |
|  | **Proportion achieving engagement** |  |
|  | **Denominator** |  |
|  | **Is there an Active User definition?** | Categorical: Yes or No |
|  | **If yes - what was the definition?** |  |
|  | **Did this definition use the digital component of the intervention?** | Categorical: Yes or No |
|  | **If yes - what indicators were used?** |  |
|  | **If yes - No. achieved this** |  |
| Primary Analysis | **Analysis Population Reported** | Categorical: ITT, Modified ITT, Per-Protocol, |
|  | **N in Analysis (Intervention)** |  |
|  | **N in Analysis (Control)** |  |
|  | **Stratification** | Categorical: Age, Gender, Site etc. etc. |
|  | **Outcome** |  |
|  | **Time of Primary Endpoint** |  |
|  | **Number Included in Primary Analysis** |  |
|  | **Analysis Method** |  |
|  | **Number at Primary Endpoint** |  |
|  | **Result (Binary)** | HEADING ONLY |
|  | **Method** | Categorical: Odds Ratio, Risk Ratio |
|  | **N with outcome (intervention)** |  |
|  | **N with outcome (control)** |  |
|  | **Result (Continuous)** | HEADING ONLY |
|  | **Baseline Mean (if adjusted for)** |  |
|  | **Mean (Continuous)** |  |
|  | **SD (Continuous)** |  |
|  | **P-value (Continuous)** |  |
|  | **Standardised Effect Size - Derived** |  |
|  | **Was primary analysis adjusted for engagement?** | Categorical: Yes or No |
|  | **Was an engagement adjustment performed?** | Categorical: Yes or No |
| **IF PRIMARY NOT ADJUSTED FOR ENGAGEMENT ABOVE COMPLETE BELOW FOR ENGAGEMENT ADJUSTED ANALYSIS (if Reported)** | | |
| Engagement Analysis | **Analysis Population Reported** | Categorical: ITT, Modified ITT, Per-Protocol, |
|  | **N in Analysis (Intervention)** |  |
|  | **N in Analysis (Control)** |  |
|  | **Analysis Method** |  |
|  | **Outcome Used** |  |
|  | **Number Included in Analysis** |  |
|  | **Engagement Adjusted Analysis Result** | HEADING ONLY |
|  | **Binary** | HEADING ONLY |
|  | **Proportion** |  |
|  | **Method** |  |
|  | **N with outcome (per arm)** |  |
|  | **Continous** | HEADING ONLY |
|  | **Baseline Mean (if adjusted for)** |  |
|  | **Mean (Continuous)** |  |
|  | **SD (Continuous)** |  |
|  | **P-value (Continuous)** |  |


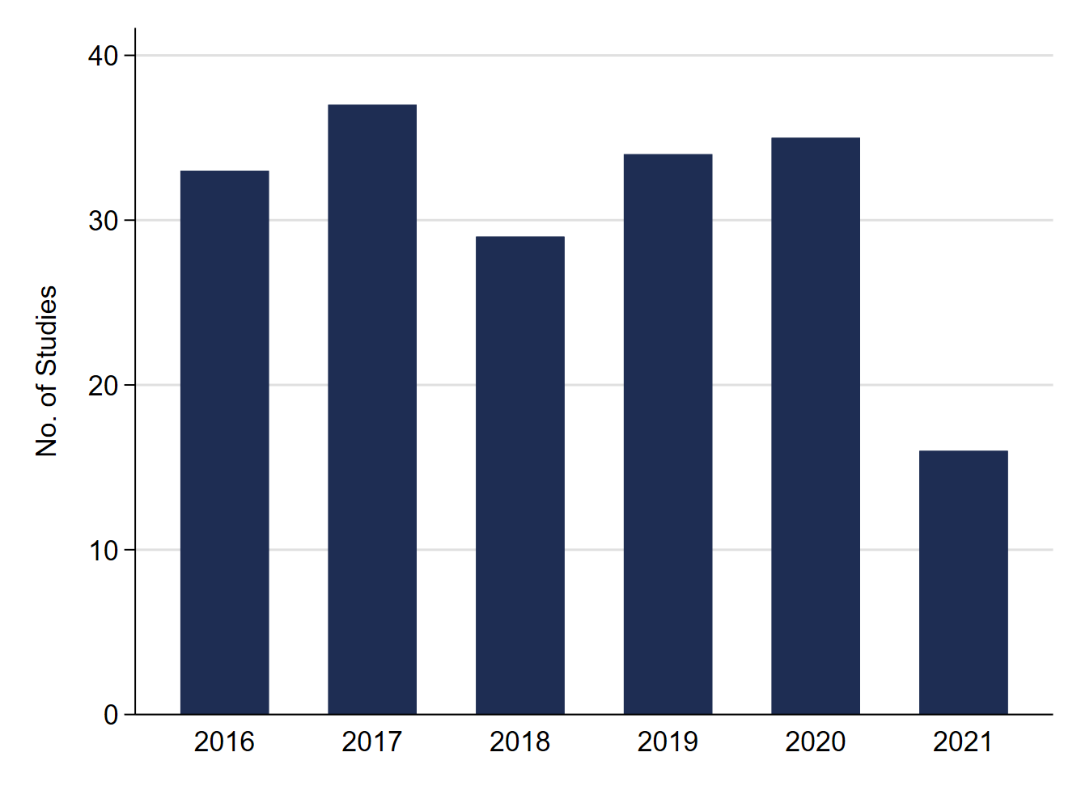


Figure S1 – The number of RCTs of DMHI in CMD publications each year


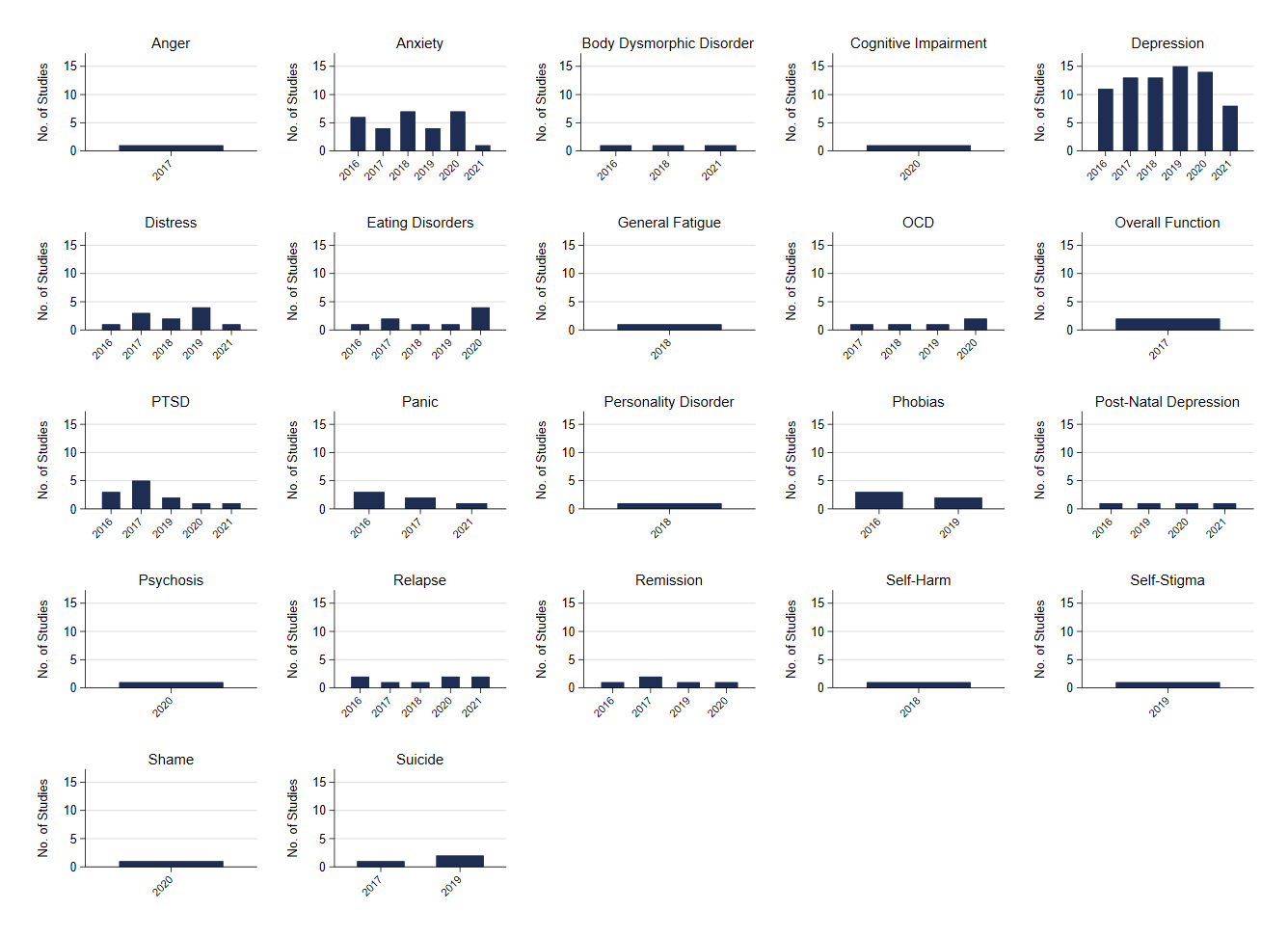


Figure S2 – The number of RCTs of DMHI in CMD publications by year for each outcome domain analysed.

Table S3 – Summary of all the health domains reported for all included trials

| **Primary Outcome Diagnosis, n studies (%)** | **N Studies** | **(%)** |
| --- | --- | --- |
| Anger | 1 | (0.5) |
| Anxiety | 29 | (15.8) |
| Body Dysmorphic Disorder | 3 | (1.6) |
| Cognitive Impairment | 1 | (0.5) |
| Depression | 74 | (40.2) |
| Distress | 11 | (6.0) |
| Eating Disorders | 9 | (4.9) |
| General Fatigue | 1 | (0.5) |
| OCD | 5 | (2.7) |
| Overall Function | 2 | (1.1) |
| PTSD | 12 | (6.5) |
| Panic | 6 | (3.3) |
| Personality Disorder | 1 | (0.5) |
| Phobias | 5 | (2.7) |
| Post-Natal Depression | 4 | (2.2) |
| Psychosis | 1 | (0.5) |
| Relapse | 8 | (4.3) |
| Remission | 5 | (2.7) |
| Self-Harm | 1 | (0.5) |
| Self-Stigma | 1 | (0.5) |
| Shame | 1 | (0.5) |
| Suicide | 3 | (1.6) |

Table S4 – Summary of the types of indicators used to summarise user engagement.

| **Indicator** | **Total Indicators** | **Used in Recommendation** | | |  | **Used in Active User Definition** | | |
| --- | --- | --- | --- | --- | --- | --- | --- | --- |
|  |  | **No** | **Yes** | **N/A** |  | **No** | **Yes** | **N/A** |
| Duration of Use | 69 (18.7) | 14 (20.3) | 7 (10.1) | 48 (69.6) |  | 19 (27.5) | 2 (2.9) | 48 (69.6) |
| Frequency of Use | 150 (40.7) | 29 (19.3) | 1 (0.7) | 120 (80.0) |  | 35 (23.3) | 6 (4.0) | 109 (72.7) |
| Milestone Achieved | 124 (33.6) | 15 (12.1) | 21 (16.9) | 88 (71.0) |  | 8 (6.5) | 24 (19.4) | 92 (74.2) |
| Communication | 26 (7.0) | 6 (23.1) |  | 20 (76.9) |  | 7 (26.9) |  | 19 (73.1) |

Table S5 – Definitions for recommended engagement levels by article identifier

| Study ID | Recommended Definition |
| --- | --- |
| Anastasiadou 2020 | Use Daily |
| Arean 2016 | Access 6 times per week for 30 minutes per day |
| Axelsson 2018 | 1 Module Per Week |
| Bucker 2019 | 1 or 2 modules per week |
| Buntrock 2016 | At least 1 session per week |
| Butler 2019 | One Lesson every 1 to 2 Weeks |
| Campos 2019 | Two Exposure Scenarios per Week |
| Christoforou 2017 | 1 or 2 sessions per week at their own pace |
| Ciuca 2018 | One or two modules per week, depending on the complexity of the content and the homework assignments |
| deKleine 2019 | All four training sessions within one week |
| Fassnacht 2018 | One Module Per Week |
| Forand 2018 | One Session Per Week |
| Hadjistavropoulos 2016 | one module per week |
| Harrer 2021 | Maximum of Two Modules per Week |
| Hedman 2016 | Work on Homework Assignments Daily |
| Heller 2020 | One Module each Week |
| Hooley 2018 | Use Daily |
| Hur 2018 | 3 Trials Per Day |
| Ivanova 2016 | One Module per Week |
| Kahlke 2019 | One Session Weekly |
| Knaevelsrud 2017 | 2 Writing Assignments a Week |
| Kohle 2021 | 1.5 Hours Per Week |
| Lehavot 2021 | Twice a week for 8 Weeks |
| Lokman 2017 | 2 to 3 hours a week for a period of at least 4 weeks |
| McCloud 2020 | Use the app at least once per week |
| Milgrom 2016 | completion of all six sessions at a rate of one session per week |
| Moeini 2019 | 2 Sessions per Week |
| Nordgreen 2016 | 4 to 6 hours per week |
| Nygren 2019 | complete one module per week |
| Oehler 2020 | complete 1 workshop per week |
| Oromendia 2016 | One Module Per Week |
| Poppelaars 2016 | complete one level (approximately 20 - 40 min) per week |
| Pots 2016 | complete one session per week |
| Radomski 2020 | complete one session per week |
| Rasanen 2016 | one module per week |
| Reins 2019 | finish at least one, but preferably two modules per week |
| Rollman 2018 | One Session every 1 to 2 Weeks |
| Schulz 2016 | one session per week |
| Smith 2017 | complete one lesson every 1–2 weeks of the program |
| Stjerneklar 2019 | complete all eight modules (and module content) in the order they appeared within the 14-week intervention period |
| Stolz 2018 | one module per week |
| Teng 2019 | three times per day |
| Timulak 2016 | complete one module per week |
| Tonning 2021 | fill in the daily selfrating |

Table S6 – Description of the definitions of an active user by article identifier

| Study | Active User Definition |
| --- | --- |
| Andersson 2020 | Attended at least 6 modules of ICBT |
| Arean 2016 | met adherence criteria for at least 2 of the 4 weeks, defined as at least three 30 minute sessions per week |
| Beevers 2017 | Activated and spent at least 60 minutes actively engaged |
| Beshai 2020 | Completed all Modules and demonstrated High Treatment Fidelity |
| Braun 2021 | Completion of at least the second last standard lesson (equivalent to at least 80%) of the assigned training |
| Bucker 2019 | Used MOOD at least once a week |
| Buntrock 2016 | Completed at least 5 of the 6 sessions |
| Cardi 2020 | At least 4 modules completed |
| Castro 2020 | 4 Completed Modules |
| Christoforou 2017 | At least 80% of Intervention (8/10 Modules) |
| DeZwaan 2017 | Logged in until week 10 |
| Ebert 2018 | 5 out of 6 Sessions Completed |
| Eimontas 2018 | Completed Self-report assessments at T2 and at least one intervention exercise. |
| Gladstone 2018 | 2 or more modules completed |
| Heckendorf 2019 | four or more of the five sessions as having followed the protocol |
| Heller 2020 | Completed the 5 Modules |
| Hoorelbeke 2016 | Completed all 10 sessions |
| Jacobi 2017 | opened at least 25% of program pages or participated in at least two one-to-one chats |
| Kenter 2016 | >=4 of 5 sessions |
| Klein 2016 | two sessions over a total duration of at least 60 min |
| Klein 2017 | two sessions over a total duration of at least 60 min |
| Kohle 2021 | Completed all 6 Modules |
| Lobner 2018 | Used at least once |
| McCloud 2020 | Used App Weekly |
| Montero-Marin 2016 | At Least 6 Sessions |
| Nilsson 2019 | those who completed more than five out of 19 self-monitoring questionnaires and commenced at least one module in myCompass |
| Oehler 2020 | participants having finished at least four workshops in the iFD tool |
| Oromendia 2016 | At least the first three modules |
| O'Toole 2019 | At Least 3 Sessions of Therapy |
| Pots 2016 | completed at least the first six sessions |
| Pult 2018 | Provided post-assessment data and logged in at least once |
| Radomski 2020 | Four or More Sessions of Breathe |
| Robichaud 2020 | completed all five lessons |
| Rollman 2018 | completed 4 or more |
| Schroder 2020 | utilized the intervention for at least 60 min |
| Spence 2017 | youth who completed (or their parent/caregiver completed) at least 3 intervention sessions |
| Wagner 2016 | complete at least 80% of the treatment modules |
